# Beyond the Inflamed Appendix: Low-Grade Appendiceal Mucinous Neoplasm—Emphasis on Macroscopic Findings

**DOI:** 10.3390/diagnostics16020315

**Published:** 2026-01-19

**Authors:** Berkenye Csonka, Ádám Ferenczi, Anita Sejben

**Affiliations:** Department of Pathology, University of Szeged, 6725 Szeged, Hungary

**Keywords:** low-grade appendiceal mucinous neoplasm, appendiceal neoplasm, pseudomyxoma peritonei

## Abstract

Low-grade appendiceal mucinous neoplasms (LAMNs) are uncommon epithelial tumors that frequently present with symptoms indistinguishable from acute appendicitis, yet may progress to pseudomyxoma peritonei with significant long-term morbidity. We report the case of a 51-year-old male who presented with periumbilical pain, nausea, and fever. Although clinical findings suggested acute appendicitis, abdominal ultrasound demonstrated cystic dilation of the appendix, raising suspicion for appendiceal mucocele. Appendectomy revealed a markedly cystic, pearly appendix with multifocal wall discontinuities and mucinous exudate, resulting in complete distortion of normal anatomy. Histological examination reflected LAMN with extra-appendiceal spread. Early postoperative reoperation due to free intra-abdominal fluid revealed pseudomyxoma peritonei. This case underscores the diagnostic challenge of LAMN, highlights the critical role of meticulous gross examination and complete embedding, and emphasizes that subtle macroscopic findings may herald clinically significant peritoneal dissemination.

**Figure 1:**
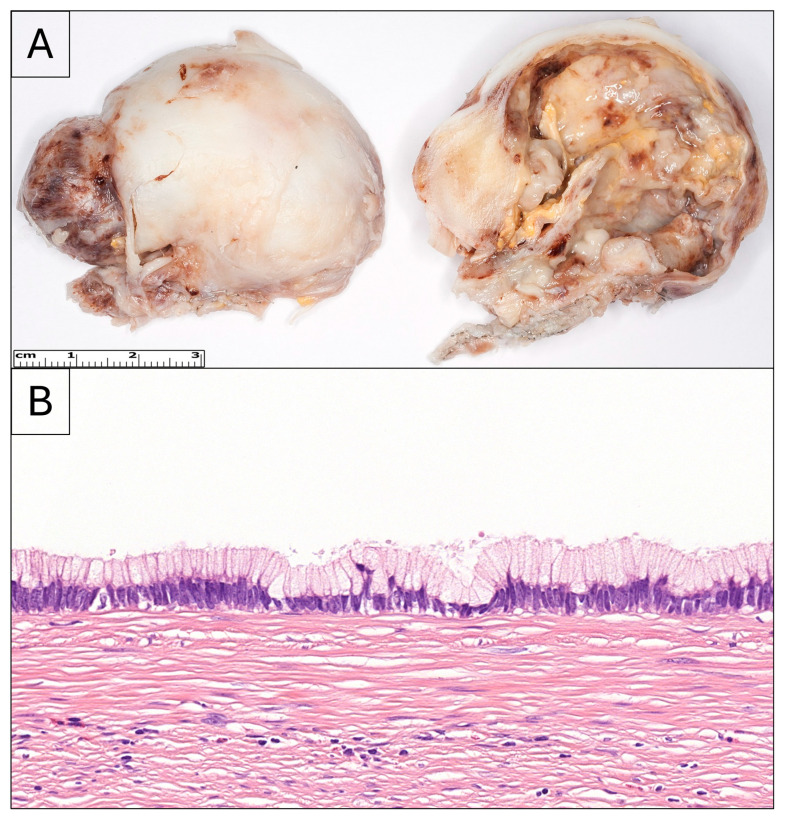
A 51-year-old male patient was admitted to the hospital due to periumbilical pain, nausea, and fever. The symptoms were consistent with acute appendicitis; however, abdominal ultrasound revealed cystic dilation of the appendix with intramural calcifications, suggestive of mucocele of 5 cm in greatest dimension. The patient was immediately operated on, and during median laparotomy, a large amount of free intraperitoneal fluid was present, with extensive serous–gelatinous deposits, suggestive of appendiceal perforation. During grossing, a cystically dilated appendectomy specimen, measuring 55 × 45 × 30 mm, was visible, with a pearly surface showing areas of discontinuity in multiple locations, with yellowish-white exudate also present (**A**). The appendix was completely distorted in shape, prohibiting proper orientation. On the cut surfaces, normal appendiceal tissue was not identifiable. In the completely embedded specimen, the appendiceal wall was edematously loosened, with minimal mucosa. Within the lumen, a flattened monolayer of mucinous epithelium was predominantly observed, with occasional filiform, multilayered, partially undulating, and partially pseudopapillary mucosal structures. Cytologic atypia was mild, with minimal mitotic activity. Furthermore, extracellular mucin was visible in the lumen and in the appendix wall. The lesion demonstrated an expansile (“pushing”) growth pattern, which manifested as transmural deposition of mucinous areas. Otherwise, the wall was fibrotic with mixed inflammatory infiltrates and regressive calcified changes ((**B**)-HE, 40×). Correlating with the macroscopic description, multiple perforations were also observed. Based on the histologic and macroscopic findings, a low-grade appendiceal mucinous neoplasm (LAMN) was diagnosed, with transmural expansile infiltration and extra-appendiceal spread. Due to a large amount of free intra-abdominal fluid with abdominal guarding, abdominal ultrasound was initiated, which revealed subhepatic and pelvic gelatinous fluid. The patient underwent reoperation four days after the initial surgery, during which a mucinous, gelatinous specimen was obtained from the vicinity of the round ligament of the liver and the adjacent liver margin. Histologically, mesothelial cells were identified embedded within abundant mucin, with occasional glandular structures exhibiting mild cytological atypia. Immunohistochemical analysis demonstrated positivity of the glandular components for KL-1 and BerEP4, findings that are consistent with pseudomyxoma peritonei (PMP) (pT4a-pM1b). Even though the lesion was incompletely excised, the patient refused both further surgical interventions and hyperthermic intraperitoneal chemotherapy (HIPEC); therefore, they have been clinically controlled with imaging since then, and no further distant metastases have been revealed. The follow-up is currently at 48 months. Based on the current WHO classification, LAMN is a mucinous neoplasm, histologically characterized by mucinous epithelial proliferation, extracellular mucin, and broad pushing margins [1]. It typically presents with symptoms mimicking appendicitis, sometimes with perforation, while peritoneal dissemination may cause abdominal distention, palpable masses, or umbilical hernias [1,2]. Imaging may show a fluid-filled appendiceal mass, occasionally with curvilinear wall calcification [1,3]. In cases of PMP, imaging may also detect deposits of mucin-density material on the peritoneal surface of abdominal viscera [4]. LAMNs most commonly occur in adults in their sixth decade and affect men and women equally [1,2,5]. The pathogenesis often involves *KRAS* and *GNAS* mutations, with rare mutations in other genes; however, typical colorectal carcinoma mutations are uncommon [1,6]. It has been suggested that the *GNAS* mutation carries significance in increased mucin production [6]. Macroscopically, the appendix may appear dilated or normal, sometimes with wall thickening, calcification, or gross perforation [1,2,7]. It has been reported that only 75% of appendiceal perforation related to LAMN is discovered during surgery; uncovering the remaining 25% relies on the meticulous grossing and microscopic examination of the pathologist [8]. Mucocele describes the gross phenomenon of cystic dilation of the appendix due to intraluminal accumulation of mucinous material; however, it can be the result of various neoplastic or non-neoplastic diseases of the appendix. A diameter of over 20 mm suggests neoplastic origin [9,10]. The uncertainty of the etiology of dilation warrants careful intraoperative handling to minimize the possibility of iatrogenic rupture, which may lead to invasive therapeutic consequences for the patient if eventually proved neoplastic [10]. It has been observed that approximately one-fourth of LAMNs present as a mucocele on abdominopelvic CT when this diagnostic modality is employed in the workup of acute abdominal pain [11]. In cases of perforation, mucin deposits are most often limited to a closed-off pocket in the periappendicular region, rather than diffusely embedding in the peritoneal cavity; although in instances of grossly visible rupture of the appendix, development of PMP is highly likely [9,12]. The presence of periappendicular mucin poses a five-fold risk of progression to PMP among perforated cases of LAMN [11]. Histologically, LAMNs show filiform or villous mucinous epithelium with tall cytoplasmic mucin vacuoles, mild atypia, fibrosis, hyalinization, and extracellular mucin dissection; they grow in a pushing, non-infiltrative pattern [1,2,13]. Ulceration is often present in LAMNs, which, if extensive, creates a diagnostic difficulty as little or no epithelium is examinable. This underlines the importance of complete embedding of the received specimen [9]. High-grade appendiceal mucinous neoplasms (HAMNs) are rare; they represent about 5% of mucinous neoplasms of the appendix, and exhibit similar architecture with high-grade cytologic features. Histologically, micropapillary and cribriform morphology and single-cell necrosis of epithelial cells may be present, and mitotic figures are often visible, including atypical mitoses [1,2,13]. However, the diagnosis of mucinous adenocarcinoma requires unequivocal evidence of invasive growth accompanied by a desmoplastic stromal reaction [1]. Differentiation among the above-mentioned histological entities is crucial, as potential routes of spread and prognosis are quite different. Mucinous adenocarcinoma carries the risk of lymphatic spread; AMNs do not show lymphatic invasion. HAMNs may have less favorable prognoses than LAMNs, but only in cases of perforation. The clinical outcome of unperforated LAMNs and HAMNs does not differ significantly [14]. The prognosis of LAMN depends on stage and patient age as well. In situ tumors carry virtually no risk of PMP progression [11]. Tumors confined to the appendix have an excellent outcome, while peritoneal dissemination carries a variable prognosis [1,15]. The cellularity of extra-appendicular mucin is a key factor; an unfavorable patient outcome is more likely if mucin deposits contain neoplastic cells, developing the aforementioned PMP [10]. Slow, yet continuous growth of implanted tumor cells and the formation of mucinous ascites are characteristic of PMP; however, it is unusual to find lymphatic metastasis or spread outside of the abdominopelvic cavity [9]. Prognosis can be improved with complete cytoreductive surgery (CRS) and HIPEC [1,13,15]. The term CRS describes the surgical removal of all macroscopically visible tumor deposits in the abdominopelvic cavity; the employment of HIPEC after CRS serves to destroy any microscopic residual tumor cells. It is worth noting that the use of HIPEC at present is not common practice and is available in select centers only [16]. Some suggest rigorous post-appendectomy surveillance in place of CRS and HIPEC [11]. PMP risk among patients diagnosed with LAMN is 4.9–23% [8,11,17,18,19]. The possible progression to PMP post surgery—be it appendectomy, cecectomy, or right hemicolectomy—is highest within the first three years, and most (75%) show at least suspicious features on MRI one year after surgery [8,11]. The average progression-free interval is 12.4 months [19]. The figure strikingly illustrates a classic yet frequently under-recognized diagnostic pitfall. The image vividly conveys how subtle-appearing gross lesions may conceal transmural mucin spread and impending peritoneal dissemination, underscoring the indispensable role of careful macroscopic examination and thorough sampling. Such extreme cystic dilation is not typical of acute appendicitis, and should therefore alert the grossing pathologist to the possibility of an underlying neoplastic or unusual process. Furthermore, it should be emphasized that the term “mucocele” refers solely to cystic dilation of the appendix. Its underlying cause may include adenoma, mucinous neoplasm, mucinous adenocarcinoma, or even non-neoplastic conditions; therefore, all potential etiologies must be carefully considered and thoroughly evaluated clinically. The absence of an obvious tumor mass further reinforces a critical teaching point: LAMNs are often diagnosed not by what is seen, but by what is lost—normal architecture. This case powerfully highlights why LAMNs deserve heightened clinical and pathological awareness: they commonly present with appendicitis-like symptoms, may appear deceptively indolent on gross inspection, yet carry the risk of lifelong morbidity once peritoneal dissemination occurs. This case also underscores that the decision to initiate HIPEC depends on the presence of perforation; therefore, LAMN specimens should be submitted in their entirety during gross examination. This case is notable in that, despite the patient’s refusal of further treatment, the observed progression-free survival exceeds the reported average more than twofold. The exceptional macroscopic documentation in the figure serves not only as a diagnostic guide, but also as a visual reminder that early recognition at the grossing table can alter staging, management, and prognosis.

## Data Availability

The original contributions presented in this study are included in the article. Further inquiries can be directed to the corresponding author.

## Abbreviations

The following abbreviations are used in this manuscript:

CRS	Cytoreductive surgery
GNAS	Guanine nucleotide-binding protein, alpha stimulating activity polypeptide
HAMN	High-grade appendiceal mucinous neoplasm
HE	Hematoxylin and eosin
HIPEC	Hyperthermic intraperitoneal chemotherapy
KL-1	KL-1 cytokeratin
KRAS	Kirsten rat sarcoma viral oncogene homolog
LAMN	Low-grade appendiceal mucinous neoplasm
PMP	Pseudomyxoma peritonei
WHO	World Health Organization
